# Vibration-based biomimetic odor classification

**DOI:** 10.1038/s41598-021-90592-x

**Published:** 2021-05-31

**Authors:** Nidhi Pandey, Debasattam Pal, Dipankar Saha, Swaroop Ganguly

**Affiliations:** Department of Electrical Engineering, Indian Institute of Technology Bombay, Mumbai, India

**Keywords:** Chemistry, Engineering, Physics

## Abstract

Olfaction is not as well-understood as vision or audition, nor technologically addressed. Here, Chemical Graph Theory is shown to connect the vibrational spectrum of an odorant molecule, invoked in the Vibration Theory of Olfaction, to its structure, which is germane to the orthodox Shape Theory. Atomistic simulations yield the Eigen-VAlue (EVA) vibrational pseudo-spectra for 20 odorant molecules grouped into 6 different ‘perceptual’ classes by odour. The EVA is decomposed into peaks corresponding to different *types* of vibrational modes. A novel secondary pseudo-spectrum, informed by this physical insight—the Peak-Decomposed EVA (PD-EVA)—has been proposed here. Unsupervised Machine Learning (spectral clustering), applied to the PD-EVA, clusters the odours into different ‘physical’ (vibrational) classes that match the ‘perceptual’, and also reveal inherent perceptual subclasses. This establishes a physical basis for vibration-based odour classification, harmonizes the Shape and Vibration theories, and points to vibration-based sensing as a promising path towards a biomimetic electronic nose.

## Introduction

The complexity of our sense of smell, its strong link to emotion and memory, and the debate about its underlying mechanism, makes it scientifically intriguing. On another side, the power of biological olfaction observed in nature makes biomimetic sensors technologically attractive. Modern-day ‘electronic noses’ (gas/vapor sensors) pale in comparison to sensitive biological ones, such as those of sniffer dogs and polar bears^1^.

In the short-term, truly biomimetic electronic noses could realize powerful sensing for several domains that impact human society immensely, namely: environment, food and agriculture, safety, and security. In the long term, these could pave the way for digitizing and transmitting olfactory (and, along the same lines, gustatory) information, as is routinely done with auditory and visual information today. The quest for biomimetic electronic nose sensors impels us to explore the relationship between the structure/properties of odorants and olfactory reception (sensing)^2^.

Olfaction is a multi-stage process, starting with the odorant molecules entering the nose and ending with the brain recognizing the odour/odorant. The olfactory epithelium in the nasal cavity contains olfactory sensory neurons (about 50 million in humans, 300 million in dogs), with ORs expressed on their cell membranes. The Nobel Prize winning work of Richard Axel and Linda Buck^2^ has established that ORs belong to the class of G-Protein Coupled Receptors. An odorant molecule could bind to multiple ORs, and vice versa, with an affinity that depends on physicochemical properties of the molecule. Binding triggers structural and electrochemical changes, which eventually lead to a change in the neuron cell potential, and the generation of an action potential (electrical signal) that communicates odour information to the brain. The mechanism underlying the aforementioned binding and triggering is still not perfectly clear and forms the basis of present-day investigation^3^.

Scientists have long speculated on two possibilities when it comes to the essential property of a molecule deciding its odor: geometric shape, and vibrational energies. The Vibration Theory, first proposed by Dyson^4^, suggests that the olfactory receptors work like chemical spectroscopes, sensing the localized vibrations of odorant molecules. The Shape Theory, which was proposed later and gained wider acceptance, states that the odorants bind to the receptors after which the receptors undergo a conformational change from inactive to an active state—a so-called *docking* or *lock-and-key* mechanism^5^. Turin revived the Vibration Theory postulating Inelastic Electron Tunneling Spectroscopy (IETS) as the mechanism for detecting vibrational energies^6^. This has positioned Olfaction as a prototypical system within the new field of Quantum Biology^7^. While the Vibration Theory has been debated vigorously^8–10^, some experiments^11–14^ do suggest that molecular vibrations play a part in the perception of odor. The *swipe card* mechanism for olfaction^15^ proposes a role for vibrational energy in addition to docking. (Complex activation mechanisms going beyond docking do occur in biology, e.g. in cancer immunology^16^. On another side, it is a standard practice in Quantitative Structure–Activity Relation (QSAR) studies for biochemical molecules, in contexts such as drug design, to use their vibrational spectra as a proxy to structure^17,18^).

**Figure 1:**
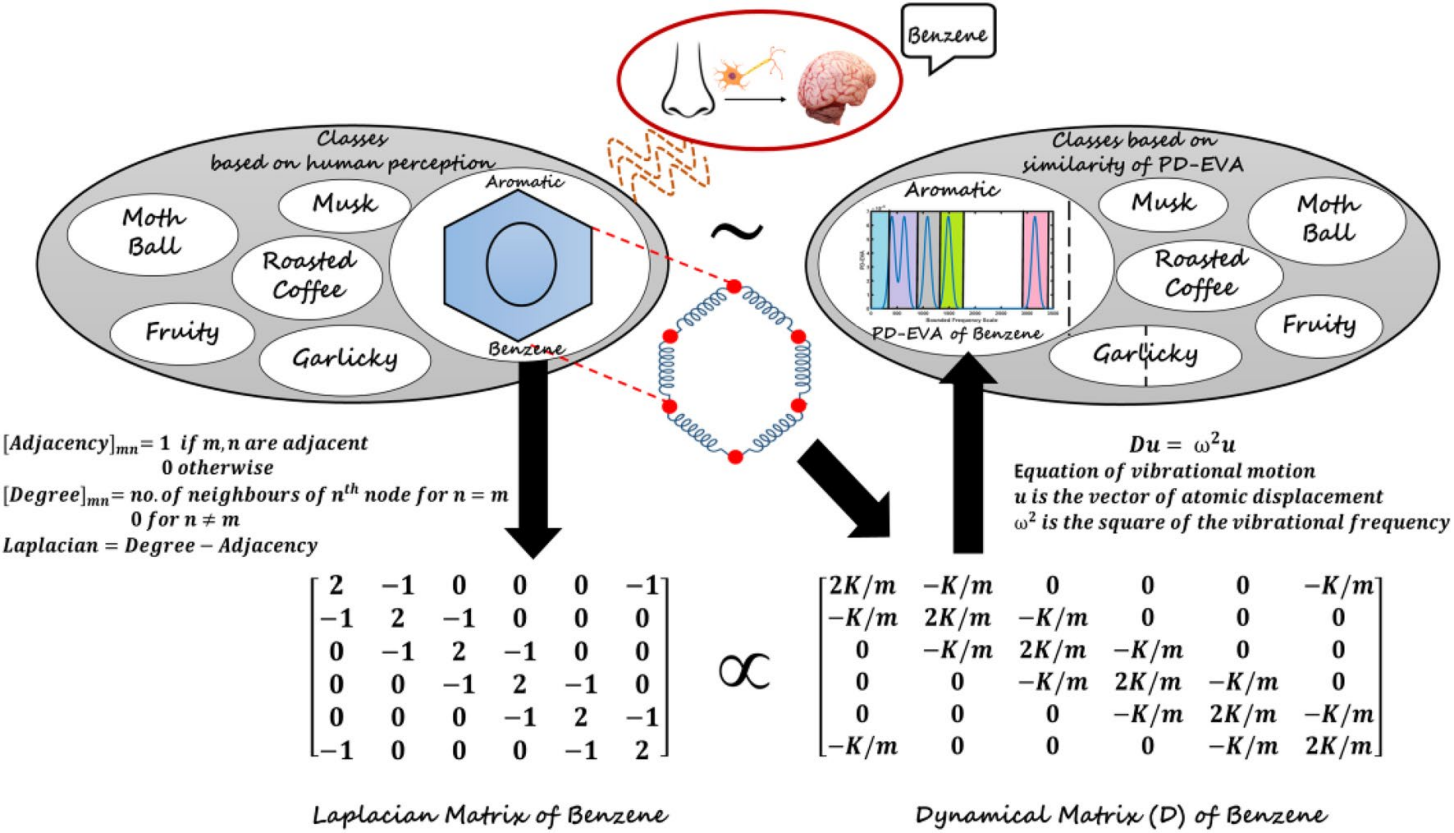
20 odorant molecules belonging to 6 different perceptual classes are first selected, including Benzene in the *Aromatic* class (left, centre). The Laplacian Matrix (left bottom), a mathematical description of the structure, is shown (for Benzene here) to be proportional to the Dynamical Matrix ${\varvec{D}}$ (right, bottom)—which describes the equation of vibrational motion of a molecule in terms of coupled oscillators. This mapping harmonizes the Vibrational and Shape theories of Olfaction. The Eigen-frequencies of the Dynamical Matrix lead to a vibrational spectrum—the Peak-Decomposed EigenVAlue (PD-EVA)—with peaks corresponding to different vibrational mode types, demarcated by frequency ranges (right, centre)—shown here for Benzene. *Clustering* based on *Similarity* in the odorants’ PD-EVA leads to the same classes as perceptual, with the revelation of subclasses within *Garlicky* and *Aromatic*. This mapping suggests that vibration-based odour sensing and classification may have the potential to emulate the power of biological olfaction.

Now, the perception of odor has been shown to be correlated with the physico-chemical properties of odorant molecules, in particular, the atomic mass distributions therein^19^. Since the mass distribution in a molecule relates both to its shape and its vibrational spectrum, this limns a path to link the lock-and-key and the vibration pictures of olfaction. Here, we seek to formally unify these two, apparently orthogonal, pictures using the apparatus of Chemical Graph Theory, and Fig. 1 demonstrates the formulation used to unify these theories. The odorant molecule is treated as a graph with the atoms as its nodes, and attached weights corresponding to relevant atomic properties. Calculation of associated matrices then relates the structure of the graph to its functional properties, such as the vibrational spectrum—which is derived from the eigenvalues of the Laplacian matrix weighted by stiffness.

Thus, we calculate the discrete vibrational spectra of 20 odorant molecules, belonging to 6 different classes, from atomistic simulations. The molecules in each class stimulate a similar perception of smell, and so these constitute our perceptual classes. The corresponding Eigen-VAlue (EVA) descriptors are then obtained by broadening and adding up all the vibrational peaks. This gives complex pseudo-spectra, which are then simplified by peak decomposition (PD) to identify the few peaks corresponding to the major vibrational modes. The centre point of these peaks constitutes a discrete spectrum again; these are broadened and added up as before to get the PD-EVA spectra. The *Similarity* measure is then calculated between each pair of these spectra. Finally, this forms the basis for *Spectral Clustering* of the odorant molecules into physical (i.e. vibrational) classes.

## Theory and method

As mentioned above, we begin by choosing a proof-of-concept dataset of 20 odorant molecules, divided into 6 classes based on human perception of their smell. These classes span odours from different walks of everyday life, namely: roasted coffee, garlic, musk, fruity, aromatic and mothball. While smell is obviously complex, and somewhat subjective, there is agreement on the dominant smell of these molecules. We note that the odorants included here also have widely differing molecular structures, shown in Table 1. Thus, we have large diversity within the confines of a small dataset.

**Table 1:**
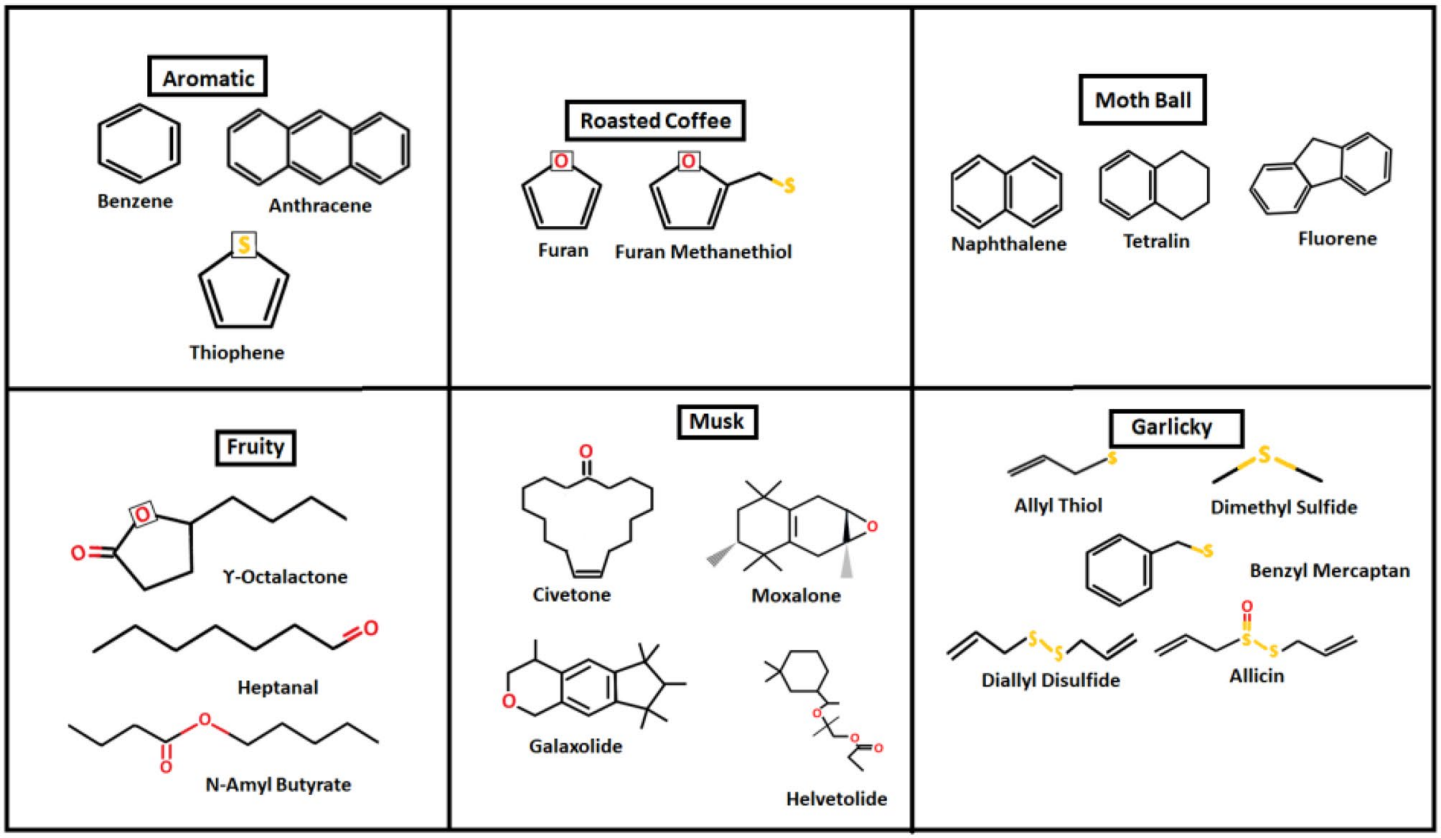
Set of odorant molecules used in this study, showing their structure, and perceptual (odour) class—namely, Aromatic, Roasted Coffee, Moth Ball, Fruity, Musk and Garlicky.

Now, the shape of a molecule may be modelled in terms of the geometry of its nuclei, and the bonds between them (understood to represent mean electron distributions). The mathematical language for this is Chemical Graph Theory, which treats the nuclei as nodes and the bonds as edges of *molecular graphs*. Then, usual graph-theoretic matrices (e.g. adjacency), suitably weighted by atomic properties (e.g. mass, electronegativity), can capture the properties of molecules^20^.

In particular, it is known that the Laplacian matrix of a molecular graph, when weighted by the ratio of the mechanical stiffness of the bonds to the mass of the atoms, yields its Dynamical matrix, whose eigenvalues and eigenfunctions comprise the vibrational spectrum of the molecule [S1]. This implies that the vibrational spectrum of a molecule is a characteristic property of its structure and constituents. A realistic, calculated rendering thereof is attempted by the construction of a pseudospectrum called the EigenVAlue (EVA) molecular descriptor, which is built upon the thesis that “a significant amount of information pertaining to molecular properties, in particular, biological activity, might be contained within the molecular vibration wave-function, of which the vibrational spectrum is a fingerprint”^18^. It has become a standard technique for similarity searching in chemical structure databases, strengthening the link between molecular structure, vibration, and activity through its empirical success.

In this work, molecular vibrational spectra have been obtained using the QuantumATK atomistic simulation package. It has an extensive materials database of molecules and crystals; and has a provision to *build* molecules that are not available in the database. In our case, Furan, Benzene, Anthracene, Naphthalene, Fluorene and Tetralin were available in the database; while the rest of the molecules were *built*. Where the molecule is *built*, its geometry has to be optimised to obtain the minimum energy configuration. This was done within the Local Density Approximation (LDA) in the ATK-DFT:LCAO calculator. The force tolerance was chosen to be 0.001 eV/Å, smaller than the default value of 0.05 eV/Å for greater accuracy. After the structure is optimized, its Dynamical Matrix is calculated, whose eigenvalues give us the vibrational energies of the molecule [^21,22^, S1]. The calculated spectra have been validated by comparison to reported experimental data [S3].

The flowchart for the calculation of the molecular vibrational spectra from the Dynamical Matrix in QuantumATK is given below.
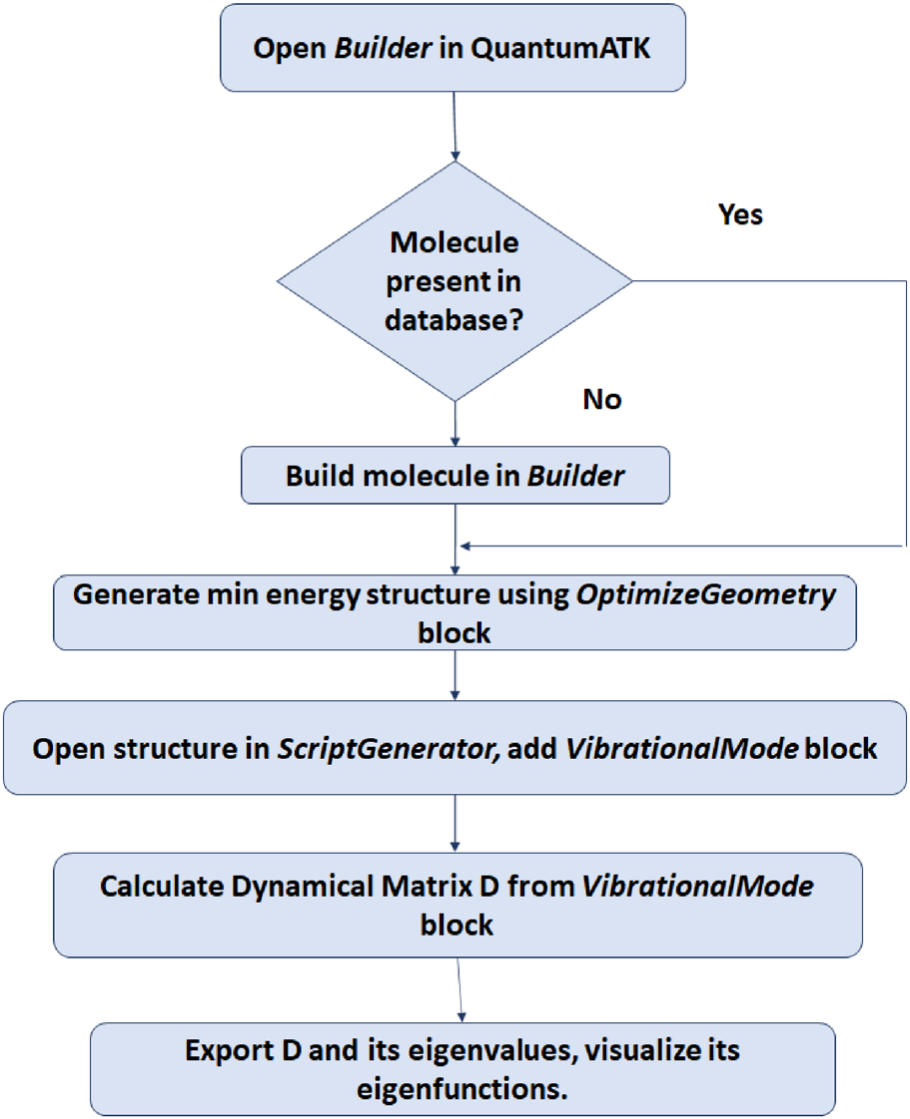


To obtain the EVA pseudo-spectrum, these vibrational modes are projected on a bounded spatial frequency (wavenumber) scale as discrete peaks of equal magnitude, and then a Gaussian kernel of appropriate standard deviation (σ) is placed on each eigenfrequency and the resulting value at any point is the sum of the amplitudes of all the Gaussian kernel at that point. The pseudo-spectrum obtained is thus:

$$ EVA\left( x \right) = \mathop \sum \limits_{i = 1}^{3A - 6} \frac{1}{{\sigma \sqrt {2\pi } }}\exp \left( { - \frac{{\left( {x - \lambda_{i} } \right)^{2} }}{{2\sigma^{2} }}} \right) $$

**Figure 2:**
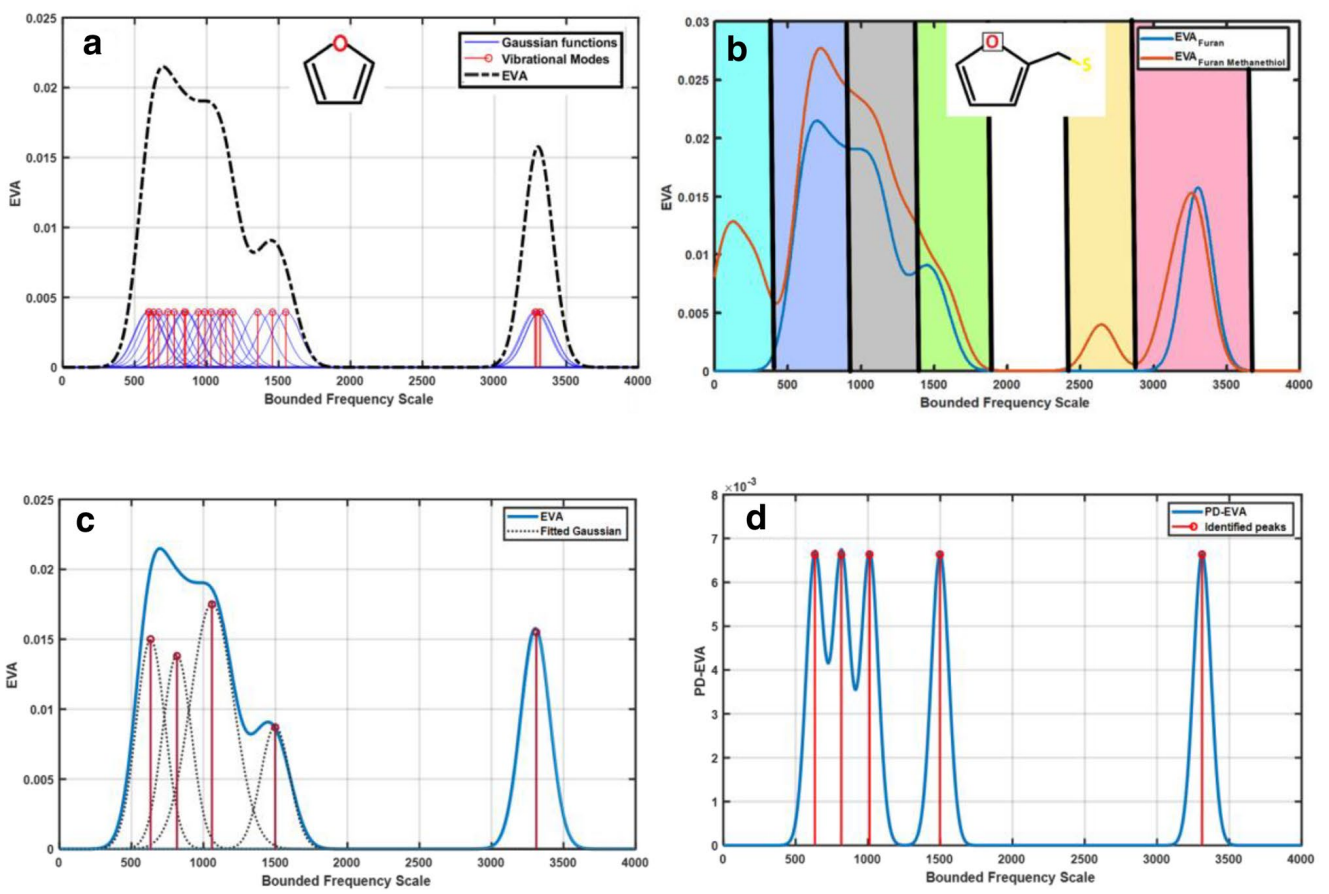
The ‘EigenVAlue’ (EVA) pseudo-spectrum for the molecule Furan (shown in inset), constructed by broadening the calculated vibrational peaks (stems in red) with Gaussian functions (in blue) of suitable σ (here 100 cm^−1^) and summing up the contributions from all of them at each frequency point**. **(**b**) The EVA pseudospectrum, of Furan Methanethiol (inset) as an example, with the partitioning of the frequency scale by types of vibrational modes. From left to right:  Torsional modes,  Ring Torsion and C–H rocking,  Ring Deformation, C–H wagging, C–C stretch,  C=C stretch, C=O stretch,  S–H stretch,  C–H stretch. Similar broad classification of modes can be done so the entire convolved EVA spectra can be deconvolved into broad peaks to identify the regions. This suggests the classification of odorant molecules by broad separation of the vibrational spectrum into these regions, accomplished through the ‘peak-decomposed EVA’. (**c**) Peak Decomposition of the EVA spectrum of Furan (shown in inset). (**d**) Construction of the Peak-Decomposed EVA (PD-EVA) spectrum by following the same procedure as for EVA (illustrated in (**a**))

Now, the EVA pseudo-spectrum is a frequency distribution plot, widely used in Graph Theory, that brings out the multiplicity of the eigenvalues of the Laplacian matrix (here, the multiplicity of vibrational modes). The Gaussian kernel spreads out the discrete eigenfrequency peaks over a standard deviation (σ) and the merges those which are close to each other on a scale of σ: thus, a smaller σ accentuates finer details like local bonding, whereas a larger value manifests its global patterns^23^. We consider here a relatively large value of σ (100 cm^−1^), corresponding to the room temperature broadening of IETS peaks, and refer to the resulting pseudo-spectra as “300 K EVA”. Figure 2a illustrates, as an example, the construction of the 300 K EVA pseudo-spectrum for Furan (the EVA pseudo-spectra for all the other molecules are shown in S3). Thermal broadening presents a well-known identification problem in IETS based sensors^24^ which have been sought to be alleviated by novel device designs^25^. Here we will see that the room temperature equivalent broadening, in fact, leads to proper odorant identification from their EVA pseudo-spectra. This suggests that: one, the “thermal broadening problem” in IETS-based sensors may not need a ‘hardware-level solution’, but can be addressed at the ‘software-level’; and two, the ‘software’ for odorant identification from vibrational peak analysis—to be presented hereafter—may, in fact, be aided by room temperature thermal broadening.

Now, it is possible to perform a classification of odorants from their EVA pseudospectra that is based purely on data science methods, and not explicitly linked to underlying physics^26,27^. In order to develop a classification scheme that is informed by physical intuition, we seek to identify the vibrational mode types, which in turn determine the frequency of the spectral peaks^28^. For example, torsional modes are typically the lowest energy ones, whereas C–H stretching modes are the highest energy. In 300 K EVA pseudospectra, discrete peaks arising from the same mode type would tend to get merged together. We follow a novel procedure to resolve the modes and generate a secondary EVA pseudospectrum carrying this information, thereby incorporating physical insight into the machine-learning based classification that follows. First, the 300 K EVA is decomposed by standard methods (using ‘Peak Deconvolution’ in the software Origin^29^) into a few broad peaks—far fewer than the number of original discrete peaks we started with. Dividing the frequency range into intervals, as shown in Fig. 2b, then allows us to identify these broad peaks with specific mode types. Thereafter we iterate the EVA procedure as shown in Fig. 2c,d, starting this time with discrete peaks (stems) positioned at the center of the above broad peaks, and using a smaller σ (60 cm^−1^) to enable finer resolution between them. This yields the secondary EVA, the PD-EVA, which is further used for spectral clustering as described below (shown for Furan in Fig. 2c, for all the other molecules in S3). We then employ a measure, common in QSAR techniques, to compare two EVA (or PD-EVA) spectra^20^. This measure, called the Similarity, is calculated as follows:

$$ S = 2*\frac{{\mathop \sum \nolimits_{i = 1}^{n} EVA1\left( i \right)*EVA2\left( i \right)}}{{\mathop \sum \nolimits_{i = 1}^{n} EVA1\left( i \right)^{2} + \mathop \sum \nolimits_{i = 1}^{n} EVA2\left( i \right)^{2} }} $$

The pairwise Similarity Indices so obtained are arranged in a n × n matrix, called the Similarity Matrix where the columns and rows correspond to the n odorant molecules under study^30^. This is then used for Cluster Analysis.

Cluster Analysis is used to group and/or search for similar objects in large databases. Here, objects grouped within the same cluster should be more alike (per some well-defined measure) than those outsides. In particular, we use a technique called Spectral Clustering, wherein a normalized Laplacian Matrix is calculated from an effective Adjacency Matrix—which is obtained from the Similarity Matrix by setting its diagonal elements to zero. The first ‘m’ eigenvalues and eigenvectors of this normalized Laplacian are then used to cluster the odorants into ‘m’ groups using the standard k-means algorithm.

The following flowchart explains the process of clustering used to classify the odorant molecules based on their PD-EVA pseudo-spectra^30^:
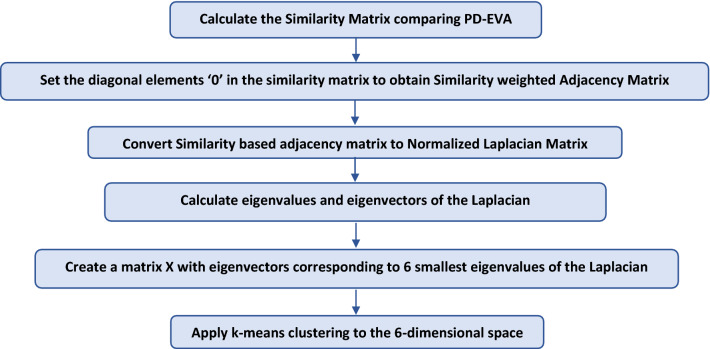


## Results and discussion

The Physics-Informed Machine Learning method described in the previous section is applied to cluster the 20 odorant molecules into physical (vibrational) classes based on the Similarity in their PD-EVA spectra. We find that optimal clustering leads to 8 physical classes, in contrast to the 6 perceptual ones (something similar was also observed earlier^26^). However, the molecules in each physical class do smell the same. It transpires that two of the six perceptual classes split into two in the process of physical (vibration-based) clustering. This will be elucidated in the following paragraph. Table 2 lists the molecules in bands indicating the physical classes, the perceptual class (i.e. smell) for each molecule, and its dominant vibrational modes obtained through peak decomposition of its EVA pseudospectrum.

**Table 2:** Molecules with their Perceptual classes and the dominant vibrational modes (in cm^−1^) identified by peak-decomposition of EVA pseudo-spectrum. A, B: Low-frequency torsional modes. C, D: Ring torsion and C–H rocking. E, F: Ring deformation, C–H wagging, and C–C stretch. G: C=C stretch. H: C=O stretch. I: S–H stretch. J: C–H stretch.

Molecule	Odor Perception	A	B	C	D	E	F	G	H	I	J
Benzene	Aromatic (Strong)			399	637		1084	1485			3189
Anthracene	Aromatic (Weak)				615	826	1147	1446			3206
Thiofuran	Aromatic (Weak)				538	758	1043	1424			3258
Furan	Roasted Coffee				633	816	1011	1498			3311
Furan methanethiol	Roasted Coffee	157			684		1025	1526		2650	3251
Naphthalene	Moth-ball	151		441		842	1150	1515			3213
Tetralin	Moth-ball	118		468		827	1147	1471			3080
Fluorene	Moth-ball	137		463		871	1126	1456			3193
Heptanal	Fruity	79				883	1287		1817		3046
N-amyl Butyrate	Fruity	118				846	1157	1386	1780		3066
ϒ-Octalactone	Fruity	112				820	1263		1816		3066
Civetone	Musk		240				1044	1377			3009
Moxalone	Musk		292				993	1370			3029
Galaxoltide	Musk		318				989	1373			3035
Helvelotide	Musk		280				999	1359			3029
Benzyl Mercaptan	Garlicky (artificial)		260			881	1091	1454		2585	3193
Allyl thiol	Garlicky (artificial)		272				927	1313	1696	2602	3086
Dimethyl sulfide	Garlicky (artificial)		226			885		1371			3051
Diallyl disulfide	Garlicky (natural)	110		439			922	1254	1724		3070
Allicin	Garlicky (natural)	120		382			958	1313	1692		3109

In fact, these classes correspond to a natural subgrouping of the perceptual class, namely ‘weakly aromatic’ and ‘strongly aromatic’^31^. The ‘garlicky’ perceptual class (last in Table 2) is also found to split into 2 physical classes; again, this is found to correspond to natural perceptual sub-classes. Allicin and diallyl disulfide occur naturally in garlic and are responsible for its odor, whereas benzyl mercaptan, dimethyl sulfide, and allyl thiol are synthetic molecules which are perceived as sulfurous-garlic^32^. It is intriguing that a purely mathematical clustering algorithm, using a descriptor originating from molecular vibrational modes, is able to resolve the perceptual subclasses. This indicates that classification and identification of odorant molecules based on intricate information about their vibrational spectra, can effectively emulate biological olfaction and enable biomimetic olfactory sensors.

## Conclusion

In conclusion, we have used Chemical Graph Theory to illuminate the link between molecular structure and vibrational spectra that is implicit in QSAR studies based on the EVA molecular descriptor. This is consistent with earlier work^19^ suggesting that, of numerous physicochemical properties, the most crucial ones contributing to the complex perception of smell are molecular mass and its distribution—which naturally connect to its vibrational spectra in the framework of Chemical Graph Theory. We have introduced a novel vibrational pseudo-spectrum called the PD-EVA, which incorporates physical insight about vibrational mode types. A small, proof-of-concept set of 20 odorant molecules, belonging to 6 perceptual classes are classified into physical (vibrational) by Spectral Clustering based on Similarity between their PD-EVA. It is found that the best clustering leads to 8 physical classes, corresponding to the perceptual ones, plus one subclass each that was inherent in 2 of the perceptual classes and revealed in the clustering process. With this mapping, PD-EVA places vibration-based odour classification on a firm physical foundation, which was missing in earlier EVA-based clustering^26^. Thereby it strengthens the thesis that Vibration plays a non-trivial role in Olfaction. It also suggests that the power of biological olfaction may be possible to emulate with vibration-based sensing and identification. Thus, our approach could pave the way toward automated odour classification and artificial odour design for applications like perfumes and cosmetics. We underline, of course, that this study bears verification with much larger odorant data sets, which must be the subject of future work. Nonetheless, we remind ourselves that biophysical models, even if simplistic or incomplete, have proven highly effective in guiding the development of useful bio-inspired technologies, e.g. learning using neural networks. This work could similarly guide the development of a quantum biomimetic electronic nose, where the path to realizing a practical vibration/IETS based sensor system^24,33^ seems clearer than it is for sensors based on many other physicochemical properties.

## Supplementary information

Supplementary Information.
