# Supplementary material for: Vibration-based biomimetic odor classification
Source: Sci Rep. 2021 May 31;11:11389. doi: 10.1038/s41598-021-90592-x (PMC8166841; doi:10.1038/s41598-021-90592-x)
Supplement: Supplementary file 1 — Supplementary Information. [file 41598_2021_90592_MOESM1_ESM.pdf]

Supplementary Information

VIBRATION-BASED BIOMIMETIC ODOR CLASSIFICATION

Nidhi Pandey, Debasattam Pal, Dipankar Saha, Swaroop Ganguly
Department of Electrical Engineering
Indian Institute of Technology Bombay, India

S1. Chemical Graphs and Molecular Vibrational Spectra

Chemical graph theory is the branch of chemistry wherein chemical objects like molecules, crystals, polymers, clusters etc. are represented as mathematical objects called graphs. A graph is a collection of nodes, and edges connecting them. For a chemical graph, the nodes correspond to atoms, and edges correspond to the bonds between them. *Molecular descriptors* are obtained as logical and/or mathematical operations on molecular graphs, transforming their chemical information into mathematical form. They are expected to correlate with physical observables measured by standard experiments.

<u>Molecular Graph</u>

For molecular graph G = (N, E), having n=[N] nodes and m=[E] edges, the nodes $v_i \in N$ represent non-hydrogen atoms and the edges $(v_i, v_j) \in E$ represent covalent bonds between the corresponding atoms. In particular, hydrocarbons are formed only by carbon and hydrogen atoms and their molecular graphs represent the carbon skeleton of the molecule. The molecular graph for benzene is illustrated below as an example.

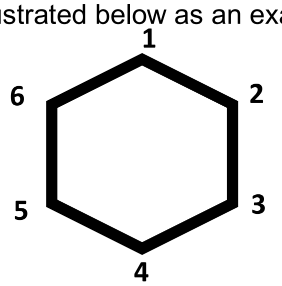

<u>Adjacency Matrix</u>

Derived from a molecular graph, the Adjacency Matrix A represents the set of connections between the adjacent pair of atoms. The entry a_{ij} is non-zero if nodes (atoms) v_i and v_j are adjacent to each other and zero otherwise.

Going by this, the Adjacency Matrix A for the benzene molecule above is seen to be:

$$A = \begin{matrix} 0 & 1 & 0 & 0 & 0 & 1 \\ 1 & 0 & 1 & 0 & 0 & 0 \\ 0 & 1 & 0 & 1 & 0 & 0 \\ 0 & 0 & 1 & 0 & 1 & 0 \\ 0 & 0 & 0 & 1 & 0 & 1 \\ 1 & 0 & 0 & 0 & 1 & 0 \end{matrix}$$

The Adjacency Matrix for a molecular graph is commonly weighted by atomic properties, such as atomic mass, or electronegativity.

<u>Degree Matrix</u>

The Degree Matrix represents the degree of a node in the molecular graph, calculated by taking the sum of all elements in a single row in the Adjacency Matrix.

$$V = \begin{matrix} 2 & 0 & 0 & 0 & 0 & 0 \\ 0 & 2 & 0 & 0 & 0 & 0 \\ 0 & 0 & 2 & 0 & 0 & 0 \\ 0 & 0 & 0 & 2 & 0 & 0 \\ 0 & 0 & 0 & 0 & 2 & 0 \\ 0 & 0 & 0 & 0 & 0 & 2 \end{matrix}$$

Laplacian Matrix

The Laplacian Matrix of a graph is defined as L = V – A, where V is the Degree Matrix and A is the Adjacency Matrix of the graph. For the graph above, the Laplacian Matrix is thus:

$$L = \begin{matrix} 2 & -1 & 0 & 0 & 0 & -1 \\ -1 & 2 & -1 & 0 & 0 & 0 \\ 0 & -1 & 2 & -1 & 0 & 0 \\ 0 & 0 & -1 & 2 & -1 & 0 \\ 0 & 0 & 0 & -1 & 2 & -1 \\ -1 & 0 & 0 & 0 & -1 & 2 \end{matrix}$$

Dynamical Matrix and Molecular Vibrational Spectrum

To derive the vibrational modes of molecule, the molecule can be treated as a network of harmonic oscillators.

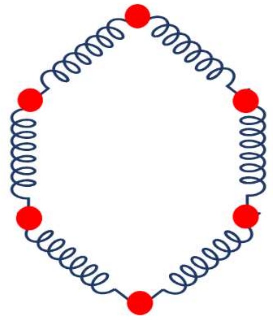

Newton's equation of motion for each atom (node) may be written as:

$$m_i \frac{d^2 u_i}{dt^2} = -\sum_{i \neq j} K_{ij}(u_i - u_j)$$

where K is the spring constant of the oscillator, m is the mass of the atoms and u_i is the displacement of the ith atom. In this section the K are assumed simplistically, for illustrative purposes, to be the same for each oscillator. The next section will elucidate how the K are evaluated in terms of interatomic forces for a realistic calculation of the vibrational modes of an actual molecule.

This can be written in the following matrix form:

$$\begin{bmatrix} \frac{K}{m} & -\frac{2K}{m} & 0 & 0 & 0 & -\frac{K}{m} \\ -\frac{K}{m} & \frac{2K}{m} & -\frac{K}{m} & 0 & 0 & 0 \\ 0 & -\frac{K}{m} & \frac{2K}{m} & -\frac{K}{m} & 0 & 0 \\ 0 & 0 & -\frac{K}{m} & \frac{2K}{m} & -\frac{K}{m} & 0 \\ 0 & 0 & 0 & -\frac{K}{m} & \frac{2K}{m} & -\frac{K}{m} \\ -\frac{K}{m} & 0 & 0 & 0 & -\frac{K}{m} & \frac{2K}{m} \end{bmatrix} \begin{bmatrix} u_1 \\ u_2 \\ u_3 \\ u_4 \\ u_5 \\ u_6 \end{bmatrix} = \omega_\nu^2 \begin{bmatrix} u_1 \\ u_2 \\ u_3 \\ u_4 \\ u_5 \\ u_6 \end{bmatrix}$$

which can be compactly expressed as:

$$\sum_j D_{ij} u^{\nu}{}_j = \omega_\nu{}^2 u^{\nu}{}_i$$

Here D is called the Dynamical Matrix and $\omega^2{}_\nu$ is the νth eigenvalue (necessarily real) which is the square of the vibrational eigenfrequency of the molecule. Comparing with the Laplacian Matrix for the above graph, we find that:

$$[D] = \frac{K}{m}[L]$$

This establishes a direct relation between the vibrational modes of a molecule – obtained through its Dynamical Matrix, and its structure – captured mathematically through its Laplacian Matrix.

References

[S1.1] Estrada, E. *Mathematical Tools for Physicists,* Wiley-VCH 2015.

[S1.2] Todeschini, R. & Consonni, V. *Handbook of Molecular Descriptors* (Wiley-VCH, Weinheim (Federal Republic of Germany), 2000).

S2. Molecular Vibrational Spectra – atomistic calculation background and flowchart

QuantumATK is a commercially available atomistic simulation software package that has been used here for the calculation of the molecular vibrational modes [S2.1]. For this, a molecule is treated as a network of quantum oscillators, of some spring constant K, connecting the massive atoms. The problem then boils down to finding the spectrum of the Dynamical Matrix as explained in the previous section. This section explains how the K emerge from the molecular Hamiltonian.

The Born-Oppenheimer (BO) approximation enables to separate the electronic and nuclear coordinates. The Hamiltonian for a molecule as an ion-electron system may be written as:

$$H = \sum_{K} \frac{{p_K}^2}{2M_K} + \sum_{K,L} V_{ion-ion}(R_K, R_L) + H_{el} + H_{el-io}$$

Here the first two terms correspond to the ionic degrees of freedom (the first term is kinetic energy). The total energy of the system for a given ionic configuration may be expressed as:

$$E_{total}[\{R_K\}, \psi_e] = \langle \psi_e | H_{el} + H_{el-ion} + V_{ion-ion}(\{R_K\}) | \psi_e \rangle$$

The solution to the Schrödinger Equation with the first two terms may be obtained through the Kohn-Sham equation in Density Functional Theory.

$$H_e = H_{el} + H_{el-ion}$$

Now, at each point on the energy surface for an ionic configuration, the ions have a potential energy E_{tota} , which is a function of ionic positions.

For the vibrational degrees-of-freedom, the Hamiltonian can therefore be written as:

$$H_{ph} = \sum_{K} \frac{{p_K}^2}{2M_K} + E_{total}[\{R_K\}]$$

Here the first terms on the right-hand side corresponds to the ionic kinetic energy as a quadratic function of their momenta, and the second to the potential energy as a function of their positions. Expanding the second term in a Taylor series around the equilibrium position:

$$H_{ph} = \sum_{K} \frac{{p_K}^2}{2M_K} + E_{total}[\{R_{K0}\}] + \sum_{K} \frac{\partial E_{total}}{\partial R_K} |_{\{R_{K0}\}} (R_K - R_{K0})$$
$$+ \sum_{K,L} \frac{\partial^2 E_{total}}{\partial R_K \partial R_L} |_{\{R_{K0}\}\{R_{L0}\}} (R_K - R_{K0})(R_L - R_{L0})$$

The first derivative of the energy will be zero in the equilibrium state. Recognizing that the second derivative is nothing but the spring constant, the Dynamical Matrix can be written as:

$$D_{KL} = \frac{1}{\sqrt{M_K M_L}} \frac{\partial^2 E_{total}}{\partial R_K \partial R_L}$$

This spectrum of the Dynamical Matrix yields the vibrational modes as described in S1 above.

[S2.1] Smidstrup S. et al., QuantumATK: An integrated platform of electronic and atomic-scale modelling tools. J. Phys.: Condens. Matter **32**, 015901 (2020)

S3. Molecular Vibrational Spectra – atomistic calculation results

The in-plane ring stretches mode of benzene, as an illustrative example.

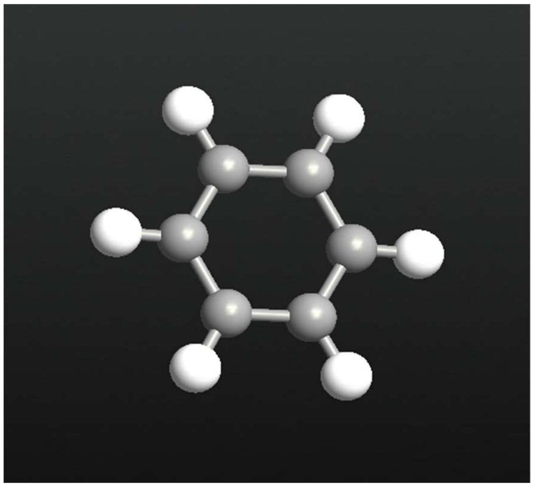

<u>Comparison to Experiment</u>

The eigenfrequencies have been compared for validation to available experimental data for two different molecules, as tabulated below.

Benzene (all modes [S3.1])

Calculated (in cm^{-1})	Experimental (in cm^{-1})
399	405
399	405
591.86	607
592.45	671
628.79	671
703.53	703
804.5	849
805.06	849
919.92	970
920.36	970
942.89	993
977.01	999
999.33	1010
1023.17	1038
1026.32	1038
1105.81	1146
1125.04	1146
1136.64	1179
1292.19	1179
1379.44	1309
1455.01	1478
1459.95	1478
1617.44	1599
1621.02	1599
3122.8	3056

3129.92	3056
3136.45	3057
3143.7	3064
3144.47	3064
3154.7	3064

Allicin (data available for select modes only [S3.2])

Mode	Calculated (in cm^{-1})	Experimental (in cm^{-1})
vS-S	449.39	474
ϒCH_2	911.62	927
ϒCH_2	963.49	989
vS=O	1076.67	1087
ρ CH_2	1237.85	1229
ρ C-H	1338.87	1319
δ CH_2	1374.84	1402
δ CH_2	1387.37	1423
v C=C	1659.76	1635
v C-H	2989	2916
v CH_2	2998	2978
v C-H	3061	3013
v CH_2	3081	3083

<u>Molecular EVA and PD-EVA</u>

The structure of all 20 molecules studied here, along with their EVA and peak decomposition (for generating PD-EVA) is shown below.

Molecule	Structure	EVA, PD-EVA
1. Furan	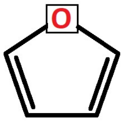	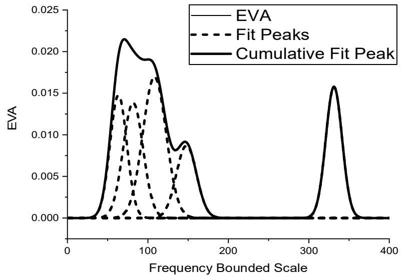
2. Furan Methanthiol	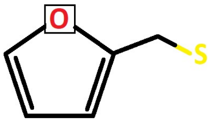	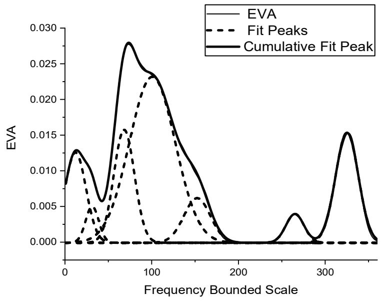

3. Naphthalene	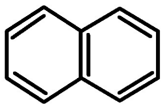	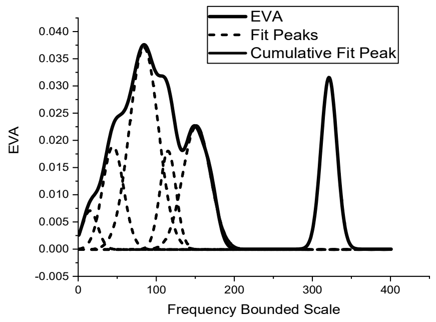
4. Tertalin	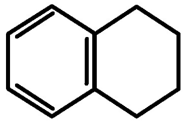	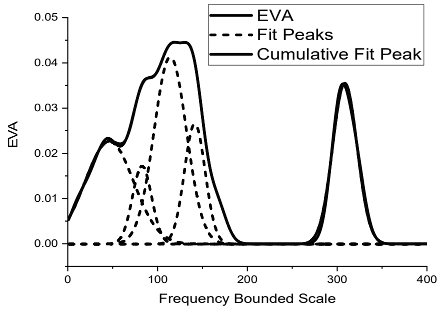
5. Fluorene	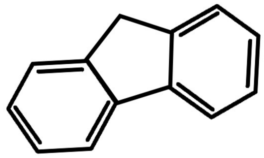	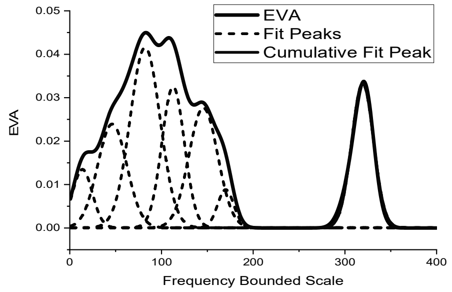
6. Benzyl Mercaptan	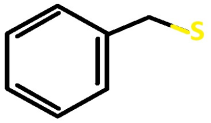	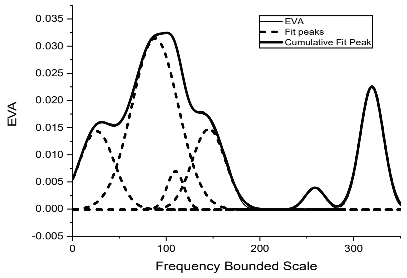
7. Allyl Thiol	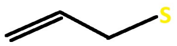	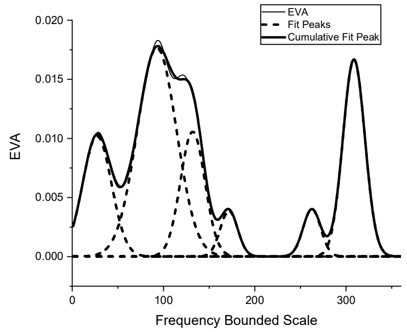

8. Dimethyl sulfide	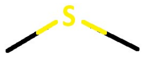	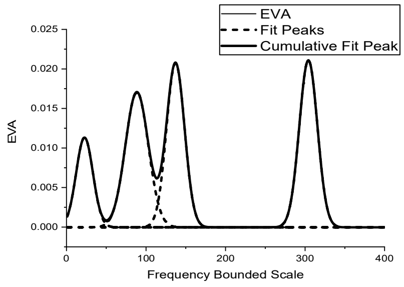
9. Diallyl disulfide	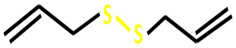	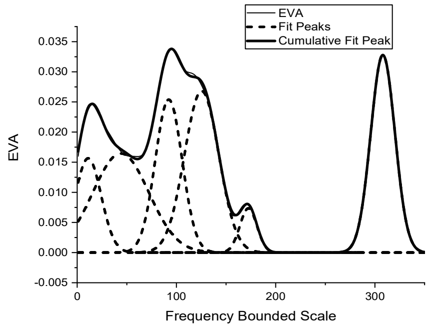
10. Allicin	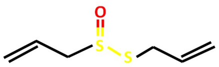	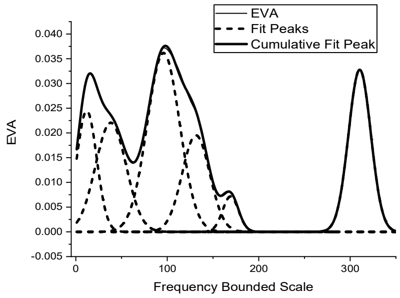
11. Benzene	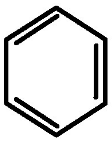	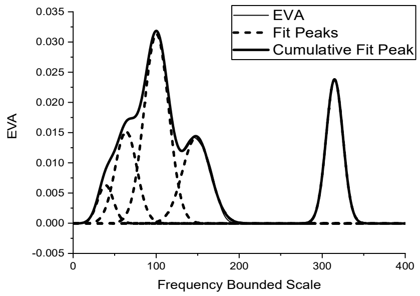
12. Anthracene	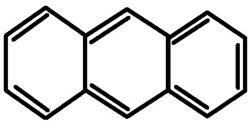	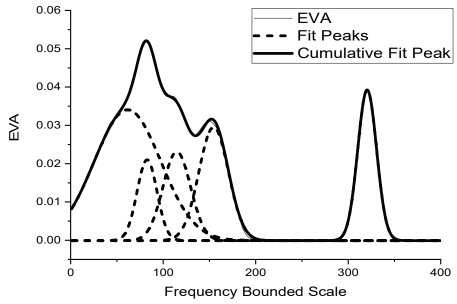

13. Thiofuran	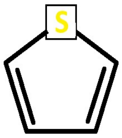	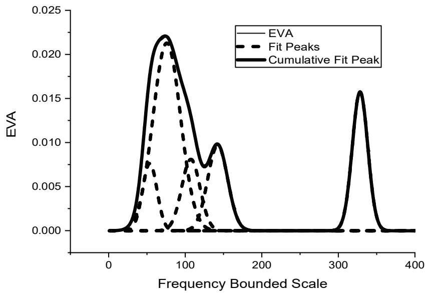
14. Civetone	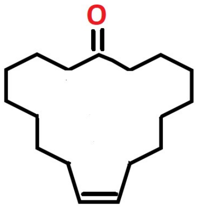	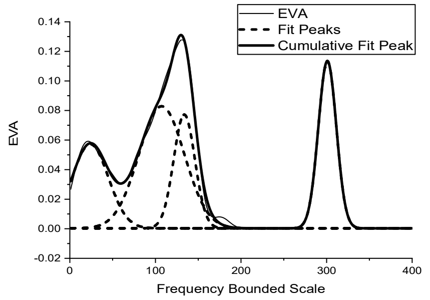
15. Moxalone	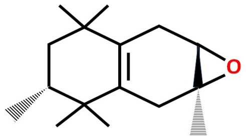	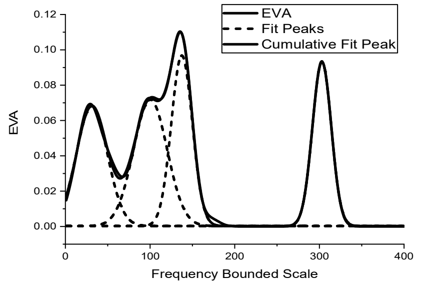
16. Galaxolide	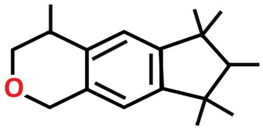	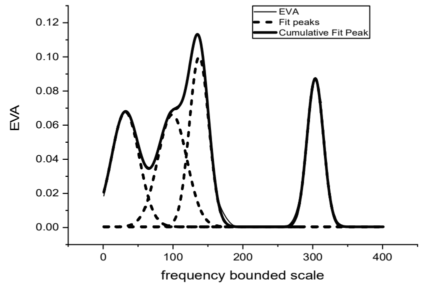
17. Helvetolide	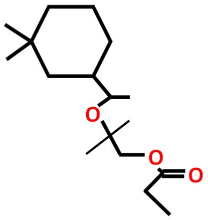	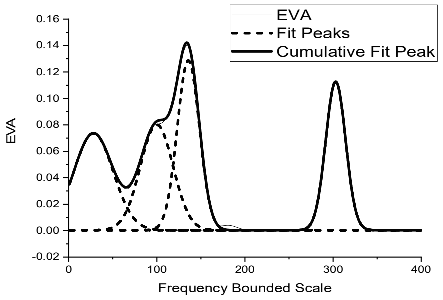

18. Heptanal	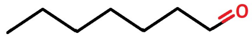	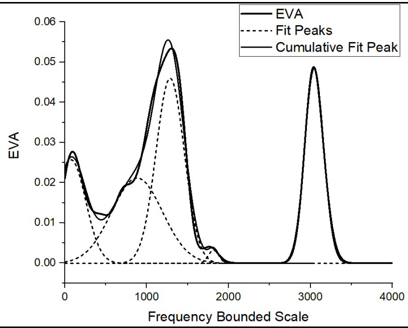
19. N-Amyl Butyrate	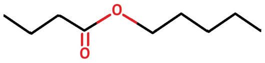	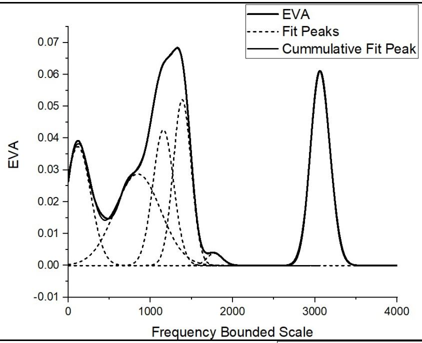
20. Ƴ-octalactone	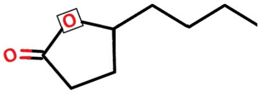	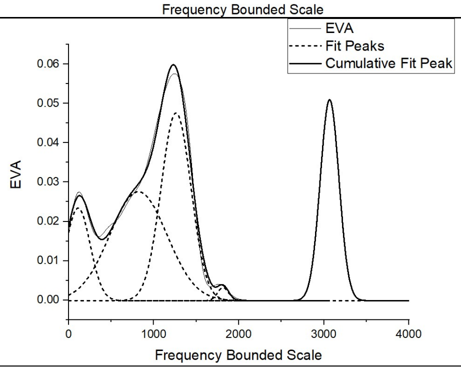

References

[S3.1] Hamdache, F., Vergoten, G., Lagant, P., & Benosman, A. Normal Modes Calculation for Benzene in a Local Symmetry Force Field. *J. RAMAN SPECTROSC.* **20**, 297-301 (1989).

[S3.2] Durlak P., Berski S., Latajka Z. Theoretical studies on the molecular structure, conformational preferences, topological and vibrational analysis of allicin. *Chem. Phys. Lett.* **644**, 5-13 (2016).
